# CNT-Supported Pt-Ni Catalysts Promoted with CeZrO_2_ and CeZrLaO_2_ for Dry Reforming of Methane

**DOI:** 10.3390/molecules31101655

**Published:** 2026-05-14

**Authors:** Mahima Kamra, Krzysztof Matus, Agata Łamacz

**Affiliations:** 1 Department of Engineering and Technology of Chemical Processes, Wrocław University of Science and Technology, 50-370 Wrocław, Poland; 2 Materials Research Laboratory, Faculty of Mechanical Engineering, Silesian University of Technology, 41-100 Gliwice, Poland; krzysztof.matus@polsl.pl

**Keywords:** dry reforming of methane, carbon nanotubes, ceria-zirconia, Pt, Ni

## Abstract

Dry reforming of methane (DRM) converts the greenhouse gases methane (CH_4_) and carbon dioxide (CO_2_) into syngas (hydrogen (H_2_) and carbon monoxide (CO)). Despite its numerous advantages, DRM has not yet been industrialized due to catalyst deactivation and competing side reactions. While Ni-based catalysts have been widely used, they are prone to increased carbon deposition and sintering, and although bimetallic systems and oxygen-based supports have shown promise, their effects on carbon deposition are yet to be fully understood. In this study, carbon nanotube (CNT)-supported Pt-Ni catalysts incorporating mixed oxides of CeZrO_2_ and CeZrLaO_2_ were investigated to evaluate the impact of support composition and metal–support interactions in DRM. The catalysts were synthesized and subsequently tested in DRM. Catalysts supported on CNTs displayed higher CH_4_ and CO_2_ conversions compared to conventional ceria–zirconia, highlighting the beneficial role of the carbon nanotube support in improving dispersion and accessibility of the metal active sites. Addition of Pt was found to promote reverse water–gas shift (RWGS) reaction, whereas the addition of La was found to decrease catalytic activity. Despite the formation of a Ni-Pt alloy, the obtained catalysts favored RWGS over DRM. These findings illustrate key limitations and design considerations for optimization of CNT-supported bimetallic catalysts in DRM.

## 1. Introduction

The continuous growth of the global population, as well as the emergence of economic powers such as China, India, and Brazil, has led to a significant increase in energy demand, which is still predominantly fulfilled by conventional fossil fuels [1]. Combustion of these fossil fuels has contributed to a sharp escalation in the emission of greenhouse gases (GHGs) such as methane (CH_4_) and carbon dioxide (CO_2_), accelerating the progression of climate change. Dry reforming of methane (DRM) is a sustainable process that has attracted significant attention since it converts these two greenhouse gases to syngas, a mixture of carbon monoxide (CO) and hydrogen (H_2_) [2]. Other reforming processes that convert CH_4_ to syngas include steam reforming (SR), autothermal reforming (ATR), and partial oxidation of methane (POM) [3]. These processes differ based on the final produced H_2_/CO ratio, with DRM having the desirable H_2_/CO ratio~1, which can be further utilized to synthesize long-chain hydrocarbons as well as other value-added products [4]. Despite the significant sustainable and environmental benefits that DRM offers, it has yet to be implemented on an industrial scale. DRM is an extremely endothermic reaction (Equation (1)) due to the intrinsic stability of CH_4_ and CO_2_ molecules, and requires high temperatures for operation (>800 °C) [5]. These high temperatures result in catalyst deactivation, resulting from either severe coke formation or active phase sintering [6]. Additionally, there are other unwanted side reactions that can occur simultaneously alongside DRM, such as reverse water–gas shift (RWGS, Equation (2)), CH_4_ decomposition (Equation (3)), Boudouard reaction (Equation (4)), and CO/CO_2_ hydrogenations (Equations (5) and (6)) [7]:

CH_4_ + CO_2_ ⇌ 2CO + 2H_2_   ΔH_298 K_ = 247 kJ mol^−1^ (1)

CO_2_ + H_2_ ⇌ CO + H_2_O   ΔH_298 K_ = 41 kJ mol^−1^ (2)

CH_4_ ⇌ C(s) + 2H_2_   ΔH_298 K_ = 75 kJ mol^−1^ (3)

2CO ⇌ CO_2_ + C(s)   ΔH_298 K_ = 172 kJ mol^−1^ (4)

CO + H_2_ ⇌ C + H_2_O   ΔH_298 K_ = −131 kJ mol^−1^ (5)

CO_2_ +2H_2_ ⇌ C + 2H_2_O   ΔH_298 K_ = −90 kJ mol^−1^ (6)

To avoid these unwanted reactions, minimize coke formation, and favor DRM, the reaction is carried out at temperatures in the range of 850–1050 °C with an equimolar feed of CH_4_-CO_2_ [8]. At these high temperatures, catalyst design becomes critical since coke formation and sintering effects can be minimized using active metals, promoters, and support materials that govern catalyst activity, stability, and resistance to deactivation [9].

Noble metals such as Pt, Rh, and Ru, as well as non-noble metals such as Fe, Co, Ni, etc., have been studied as active catalysts for DRM. Among these catalysts, Ni has been widely used for its low cost, high abundance, and high intrinsic activity for C-H bond activation. However, its application for DRM is limited owing to rapid deactivation through carbon deposition (Equations (3)–(6)) and sintering at high temperatures. Although several solutions have been proposed for these issues, stability without compromising activity is still a major concern. In contrast, noble metal catalysts generally exhibit superior resistance to coking and sintering, which is attributed to their typically smaller particle sizes and enhanced dispersion; nevertheless, their high cost significantly restricts large-scale industrial application [10]. In addition, it has been found that Ni nanoparticles with a diameter between 2 and 3 nm show enhanced resistance to carbon deposition as well as catalytic activity between 500 and 600 °C. In contrast, the amount of coke deposited is directly proportional to the size of the Ni particles. As a result, metal dispersion and particle size need to be controlled during the catalyst preparation stage [11].

Although monometallic Ni catalysts exhibit high activity in DRM, their practical application is limited by rapid deactivation from sintering and coke deposition. In order to overcome these limitations, bimetallic catalysts containing secondary metals like Ru [12], Rh [12], Co [13], Fe [14], and Cu [15], among many others, have been studied. Among these systems, the use of Ni-Pt bimetallic catalysts has been found to be promising [16] and facilitates the removal of carbonaceous species from the catalyst surface, which may result in improved catalyst stability, resistance to coking, and better metal dispersion [17]. Although the addition of Pt has been reported to suppress the reverse water–gas shift (RWGS) reaction in some oxide-supported systems [17,18], its influence on reaction selectivity remains complex, with emerging studies indicating that Pt may also promote RWGS under specific conditions. In addition to the metal composition, the approach used in incorporating the metals into the catalyst has been found to be very important in determining the final metal dispersion, metal–support interaction, and overall catalytic activity.

Another approach that has been used to improve the stability of Ni-based catalysts is the selection of the support material that can take an active role in the catalytic reaction. Catalyst supports influence performance by inhibiting metal sintering and suppressing carbon deposition, often through confinement effects and metal–support interactions [19]. Commonly used supports include metal oxides such as Al_2_O_3_ [20,21], SiO_2_ [22], TiO_2_, ZrO_2_ [23], and CeO_2_ [24]. Of these metal oxides, cerium oxide (CeO_2_) is of significant interest owing to its high reducibility, high oxygen storage capacity due to the reversibility of the Ce^4+^/Ce^3+^ redox couple, and high oxygen mobility, which helps in the activation of CO_2_ and oxidation of carbon species [25,26,27]. For Pt/CeO_2_ catalysts, strong metal–support interactions have been reported to enhance the adsorption and activation of both CH_4_ and CO_2_ at temperatures as low as 500 °C, highlighting the critical role of interfacial effects in DRM catalysis [28].

Although CeO_2_ is structurally stable at high temperatures, exposure to DRM conditions leads to sintering and reduced oxygen mobility, resulting in depletion of accessible oxygen vacancies and a loss of effective oxygen storage capacity. To improve its thermal stability and redox performance, CeO_2_ is frequently doped with thermally stable zirconium to form cerium–zirconium mixed oxides (CeO_2_-ZrO_2_, also denoted as CeZrO_2_) [29,30]. The additional oxygen vacancies and increased lattice oxygen mobility lead to substantial improvements in the oxygen storage capacity of the mixed oxide. The addition of Zr inhibits sintering of Ce at high temperatures, which results in an enhanced catalytic activity. These combined effects facilitate more efficient redox cycling and carbon removal during high-temperature reactions, ultimately reducing coke formation and enhancing catalyst stability under DRM conditions [31,32,33,34,35].

Basic oxides such as La_2_O_3_ are also attractive catalyst components due to their ability to enhance CO_2_ adsorption and activation through acid–base interactions between surface basic sites and acidic CO_2_ molecules, thereby suppressing carbon deposition and improving catalyst stability [36]. The incorporation of La_2_O_3_ alongside Al_2_O_3_ as a support for Ni catalysts has been reported to reduce Ni deactivation compared to Al_2_O_3_ alone, as La_2_O_3_ promoted stabilization of the active metallic Ni^0^ phase [37]. However, an excessive amount of La_2_O_3_ has shown negative effects due to the strong interaction between La_2_O_3_ and NiO, which may cause the formation of LaNiO_3_ with decreased catalyst activity [38]. These contrasting results indicate that incorporation of La as a promoter must be carefully optimized, as improvements in oxygen mobility do not directly correlate with enhanced catalytic performance.

The interaction between metal nanoparticles and the support material plays a critical role in determining catalytic activity, selectivity, and stability. Among these interactions, strong metal–support interaction (SMSI) has been widely recognized as a key phenomenon influencing supported metal catalysts. SMSI is typically associated with reducible oxide supports, including TiO_2_, CeO_2_, Nb_2_O_5_, and ZrO_2_, which can undergo partial reduction under pre-treatment or reaction conditions. These interactions reduce metal particle sintering and can alter interfacial electronic properties, thereby enhancing catalyst stability. Oxygen vacancies in reducible support materials are responsible for CO_2_ adsorption and activation, while interfacial metal sites promote CH_4_ dissociation, thus strengthening the bi-functional mechanism [39,40].

Carbon nanotubes (CNTs) are widely used as a catalyst support material due to their unique properties, such as a high surface area, distinctive electrical properties, and structural stability. These properties are beneficial for metal nanoparticle dispersion with minimal metal sintering effects. In recent years, CNT-supported metal catalysts have also been explored for dry reforming of methane (DRM), where improved metal dispersion and carbon tolerance are critical for mitigating catalyst deactivation [41]. CNTs also possess high thermal resistance and uniform pore size distribution, as well as chemical inertness, which makes them highly desirable as supports [42]. Figueira et al. [43] functionalized CNTs using nitric acid, which introduced additional oxygen-containing surface groups. This functionalization facilitated the incorporation of metal particles into the CNT structure. Despite the promising characteristics of CNTs as a support, the combined influence of CNTs with reducible oxide phases and bimetallic active phases remain insufficiently understood, particularly in terms of reaction selectivity and competing pathways such as RWGS. Although Pt-Ni/CeZrO_2_ has already been well established in DRM, the incorporation of carbon nanotubes is for the purpose of enhancing active site dispersion and, consequently, catalyst activity and stability. As CNTs provide high surface area and improved distribution of metal active sites, they can influence electronic interactions between the support and the active phases. The combination of CNTs and the oxides creates a hybrid support structure where the surface properties of CNTs and the oxygen storage capacity of the oxides may improve resistance to carbon deposition during DRM.

Despite extensive individual studies on Ni-based, bimetallic, and oxide-supported catalysts, a comprehensive understanding of the combined effect of CNT support, mixed-oxides, and Pt-Ni bimetallic interactions under CO_2_-rich DRM conditions is lacking. The effects of these components on catalytic activity and stability, as well as reaction pathways of DRM (and RWGS), remain to be fully understood. Hence, in this study, catalysts Pt-Ni-CeZrO_2_ and Pt-Ni-CeZrLaO_2_ supported on CNTs were synthesized and tested in DRM. By comparing these catalysts with conventional oxide-supported Pt and Ni catalysts, this study aims to provide a preliminary understanding of the effects of metal–metal interactions, support effects, and interactions between the metals and the support on the catalytic activity and stability in DRM.

## 2. Results and Discussion

### 2.1. Catalyst Characterization Results

**Figure 1:**
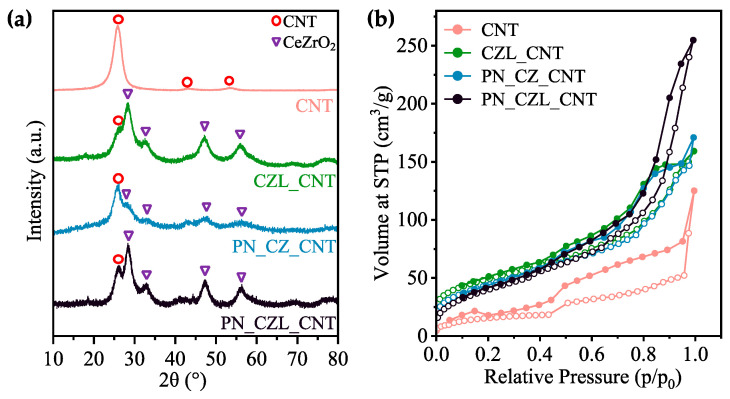
(**a**) X-ray diffractograms (XRD) of functionalized carbon nanotubes (CNT), and catalysts: CeZrLaO_2_/CNT (CZL_CNT), Pt-Ni-CeZrO_2_/CNT (PN_CZ_CNT), and Pt-Ni-CeZrLaO_2_/CNT (PN_CZL_CNT). The circles indicate the characteristic peaks of the CNTs, and the triangles correspond to the CeZrO_2_ phase. (**b**) N_2_ sorption isotherms of the catalysts measured at 77 K (adsorption—solid circle, desorption—hollow circle).

The results of X-ray diffraction (XRD) and low-temperature N_2_ adsorption–desorption measurements for functionalized CNTs (CNT), Pt-Ni/CeZrO_2_/CNT (PN_CZ_CNT), CeZrLaO_2_/CNT (CZL_CNT), and Pt-Ni/CeZrLaO_2_/CNT (PN_CZL_CNT) are shown in Figure 1 and Table 1. From the XRD patterns of the studied materials (Figure 1), the high-intensity diffraction peak observed at 25.9°, along with the low-intensity peaks at 43.5° and 53.6°, are assigned to the (002), (101), and (004) crystalline planes of graphitic carbon in CNTs, respectively [44,45]. These reflections are consistent with hexagonal graphite (JCPDS 41-1487). The peak broadening and slight shifts relative to the standard card can be attributed to the turbostratic structure and inherent defects of the CNTs. The diffractograms of CZL_CNT, PN_CZ_CNT, and PN_CZL_CNT show reflections at 28.4°, 32.7°, 47.3°, and 56.3° corresponding to (111), (200), (220), and (311) planes of the cubic structure of CeZrO_2_. During the synthesis of CeZrO_2_ phase, the substitution of Ce^4+^ by Zr^4+^ in the fluorite CeO_2_ lattice results in lattice contraction, owing to the smaller ionic radius of Zr^4+^ (the ionic radius of Ce^4+^ is 0.97 Å, and Zr^4+^ is 0.84 Å [46]). Consequently, the diffraction peaks shift toward higher 2θ angles in the XRD as compared with pure CeO_2_ structure (JCPDS 34-0394), indicating successful incorporation of Zr and the formation of a homogeneous Ce_1−x_Zr_x_O_2_ solid solution. On the contrary, lanthanum-containing samples, i.e., CZL_CNT and PN_CZL_CNT, demonstrate a slight shift in the maxima of reflections corresponding to (200) and (311) planes to lower values of 2θ, which can be attributed to the presence of stress caused by the incorporation of bigger La^3+^ cations (the ionic radius of La^3+^ is 1.16Å [47]) to CeZrO_2_. In addition, the absence of distinct diffraction peaks attributable to ZrO_2_ suggests that no phase separation between CeO_2_ and ZrO_2_ has occurred.

Due to the very low loadings of Pt, Ni, and La, the diffractograms for CZL_CNT, PN_CZ_CNT, and PN_CZL_CNT do not show diffraction peaks corresponding to these phases. As summarized in Table 1, the average crystallite size of CeZrO_2_ and CeZrLaO_2_, calculated for the (111), (200), (220), and (311) planes, is reported for all catalysts. The CeZrLaO_2_ crystallites exhibit the largest size in the PN_CZL_CNT catalyst. The increase in crystallite size upon Pt and Ni addition may be attributed to enhanced crystallite growth during thermal treatment, possibly facilitated by the presence of metal species, which promote sintering and structural ordering.

The N_2_ isotherms (Figure 1b) correspond to type IV isotherms according to IUPAC classification, indicating the presence of mesoporous structures. The initial steep uptake at low relative pressure (p/p_0_ < 0.1) is attributed to adsorption in micropores and/or on high-energy surface sites. As the relative pressure increases, a gradual increase in adsorbed volume is observed, corresponding to multilayer adsorption. At higher relative pressures, a sharp uptake is evident due to capillary condensation within mesopores. The isotherms exhibit an H4-type hysteresis loop, characteristic of narrow slit-like pores and materials containing both micro- and meso-porosity. The desorption branch shows a characteristic shoulder at p/p_0_ ≈ 0.4, which may be associated with pore network effects or cavitation phenomena.

The adsorption–desorption branches of the CNT isotherm show slight irregularities and incomplete closure at low relative pressure, which may be attributed to the presence of the micropores and the heterogeneous adsorption sites from the functionalized CNT surface. Such deviations between the adsorption and desorption branches are commonly associated with diffusion limitations within narrow pores. When the CNTs are modified with oxides and then metals, the structure may become more uniform and demonstrate lower deviation between the two branches [48,49].

Functionalized CNTs exhibit a BET surface area (S_BET_) of only 58 m^2^/g (Table 1), which increases to 176 m^2^/g after deposition of CeZrLaO_2_ nanocrystallites, likely due to the formation of additional accessible surface and modification of the pore structure. The BET surface area subsequently decreases upon the addition of Pt and Ni, which may be attributed to partial coverage of the CeZrO_2_ (CZ) or CeZrLaO_2_ (CZL) phases by metal species. Moreover, XRD analysis (Table 1) indicates an increase in CeZrO_2_ or CeZrLaO_2_ crystallite size after Pt and Ni incorporation, suggesting sintering of those oxides.

**Figure 2:**
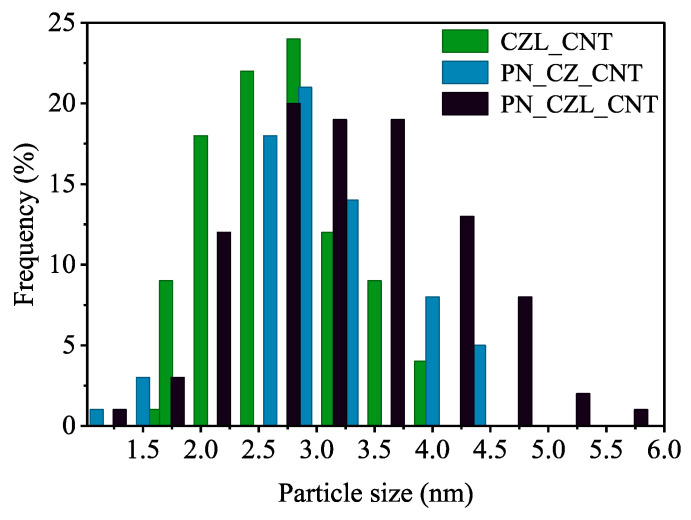
Histogram of the particle size distribution of the crystallites in CNT-supported catalysts.

Scanning and transmission electron microscopy (SEM and TEM) were employed to investigate the morphology of the catalysts. SEM images of CNT, CZL_CNT, PN_CZ_CNT, and PN_CZL_CNT are shown in Figure S1. From the microscopic observations, the CNT-supported catalysts exhibit a fibrous morphology and are composed of finely dispersed nanoscale domains of deposited oxides and metals rather than large, dense particles. The active phases containing Ce, Zr, and La are distributed along the CNT filaments, forming a porous network. Their close spatial proximity suggests intimate contact between oxide phases and the CNT support, which is favorable for interfacial interactions. The active species appear relatively uniformly distributed over the CNT surface, with no clear evidence of extensive phase segregation, although localized agglomeration cannot be excluded. The well-formed nanocrystalline domains are observed on TEM pictures. The particle size distribution (Figure S2) confirms the presence of nanoparticles. The PN_CZ_CNT sample exhibits particle sizes in the range of 1.15–4.78 nm with a mean size of 2.9 nm, while PN_CZL_CNT shows a broader distribution (1.27–6.28 nm) with a mean size of 3.26 nm, as shown in Figure 2. The CZL_CNT sample presents sizes between 1.33 and 6.16 nm, with an average of 3.13 nm. These results indicate that the active phases are predominantly present as nanoparticles, with some tendency toward agglomeration, particularly in the Pt-Ni-containing samples. The observed particle sizes are consistent with the crystallite sizes estimated using the Scherrer equation (Table 1), with PN_CZL_CNT exhibiting the largest crystallites, followed by PN_CZ_CNT, and CZL_CNT showing the smallest. This agreement suggests that the nanoparticles observed by microscopy correspond closely to the crystalline domains, and that the increase in particle size upon Pt-Ni incorporation may be associated with partial sintering and/or particle growth.

**Figure 3:**
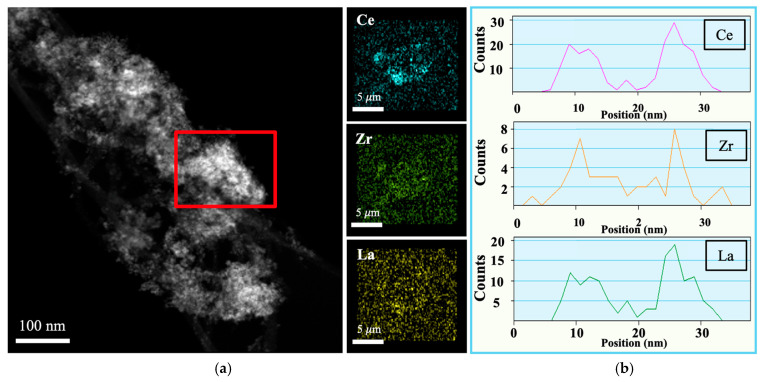
(**a**) Elemental mapping and (**b**) EDS for CZL_CNT in selected area (red rectangle).

**Figure 4:**
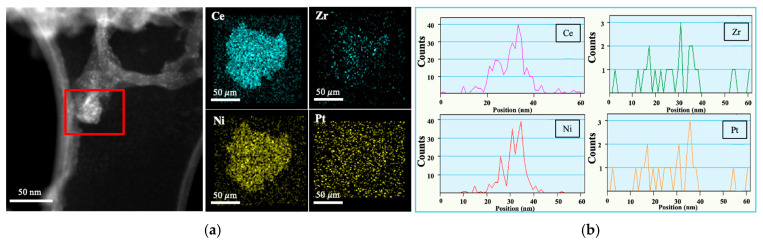
(**a**) Elemental mapping and (**b**) EDS for PN_CZ_CNT in selected area (red rectangle).

**Figure 5:**
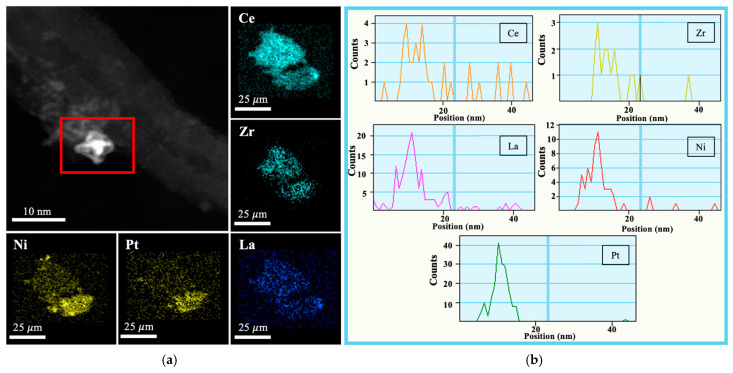
(**a**) Elemental mapping and (**b**) EDS for PN_CZL_CNT sample in selected area (red rectangle).

TEM and SAED images of CZL_CNT, PN_CZ_CNT, and PN_CZL_CNT are shown in Figures S3–S5, while elemental mapping and EDS analyses are shown in Figure 3, Figure 4 and Figure 5. For all catalysts, active phases with dimensions below 10 nm are dispersed uniformly over CNTs (Figures S3–S5). In addition, the selected area electron diffraction (SAED) confirmed the presence of CeZrO_2_ in PN_CZ_CNT and CeZrLaO_2_ in CZL_CNT and PN_CZL_CNT catalysts. SAED analysis revealed diffraction rings that could be attributed to Ni-Pt-containing phases, with lattice spacings consistent with the (110), (111), and (021) reflections of the Ni-Pt alloy structure, indicating possible formation of Ni-Pt alloy nanoparticles. Elemental mapping and EDS profiles (Figure 3, Figure 4 and Figure 5) show that Ni and Pt phases occur in close proximity, as do Ce, Zr and La. Elemental mapping for CZL_CNT and PN_CZL_CNT reveals that Ce, Zr, and La are largely co-localized within the same regions, indicating the formation of a mixed oxide phase. Their distributions overlap significantly, suggesting a homogeneous incorporation of La into the CeZrLaO_2_ oxide framework. For PN_CZ_CNT and PN_CZL_CNT, Ni and Pt are also detected within the same general region; however, their distributions appear more localized compared to the oxide components. Ni shows a broader spatial distribution with partial overlap with Ce-rich areas, indicating a tendency to associate with cerium-containing domains. In contrast, Pt is more narrowly distributed, forming concentrated regions that partially overlap with Zr-rich areas.

**Figure 6:**
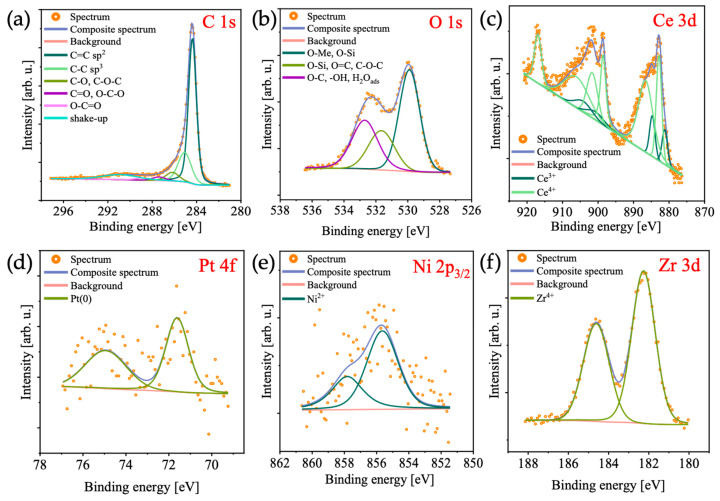
(**a**) C 1s, (**b**) O 1s, (**c**) Ce 3d, (**d**) Pt 4f, (**e**) Ni 2p, and (**f**) Zr 3d XPS spectra of the PN_CZ_CNT.

**Figure 7:**
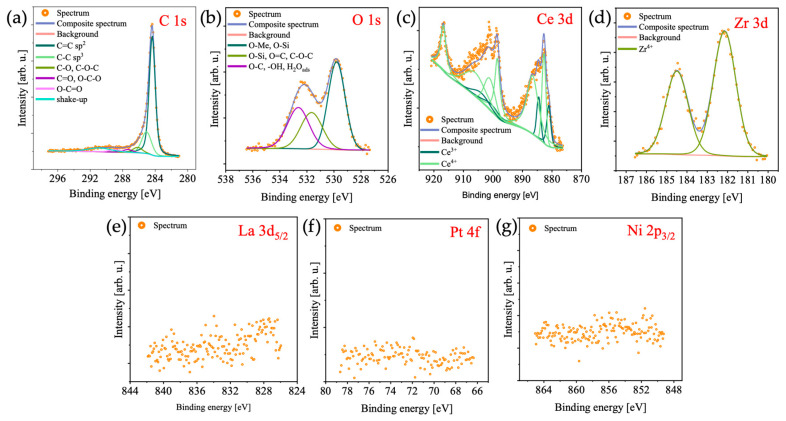
(**a**) C 1s, (**b**) O 1s, (**c**) Ce 3d, (**d**) Zr 3d, (**e**) La 3d, (**f**) Pt 4f, and (**g**) Ni 2p XPS spectra of PN_CZL_CNT catalyst.

XPS analyses for PN_CZ_CNT (Figure 6) and PN_CZL_CNT (Figure 7) catalysts were performed to investigate their surface chemical states. The C 1s spectra for both samples was deconvoluted into six components: sp^2^-hybridized C=C bonds (binding energy at 284.4 eV), attributed to graphitic carbon in the CNT network; sp^3^-hybridized C-C bonds (285.0 eV) associated with defects in the CNT framework; C-O-C and/or C-OH bonds (286.2 eV); C=O and/or O-C-O bonds (287.4 eV), corresponding to surface oxygen-containing groups; O-C=O bonds (288.8 eV), related to oxidized carbon species; and a shake-up satellite feature (290.9 eV), typically associated with graphitic materials. The O 1s spectra were fitted with three components: lattice oxygen associated with metal oxides such as CeO_2_-ZrO_2_ (530.6 eV); defective oxygen species related to oxygen vacancies or non-stoichiometric metal oxides (532.2 eV); and C-O and/or -OH groups (533.3 eV), which may also be attributed to adsorbed water. The Zr 3d XPS spectra were fitted with a characteristic doublet structure (d_5/2_ − d_3/2_), with the main 3d_5/2_ peak centered at 183.1 eV, indicative of the Zr^4+^ oxidation state, typically associated with ZrO_2_. The Ce 3d spectra revealed the presence of both Ce^3+^ and Ce^4+^ species. The spectra were deconvoluted into five doublets, corresponding to two Ce^3+^ and three Ce^4+^ contributions. The coexistence of Ce^3+^ and Ce^4+^ is indicative of oxygen vacancies, which are known to enhance oxygen mobility and can contribute to catalytic activity. A weak Pt 4f signal was detected for PN_CZ_CNT. The corresponding spectrum was deconvoluted into a doublet (4f_7/2_ − 4f_5/2_), with the main 4f_7/2_ component located at 71.4 eV, indicating the presence of metallic platinum and suggesting successful reduction in Pt species. The Ni 2p_3/2_ spectrum for PN_CZ_CNT was fitted with two components: the first, at 855.7 eV, is indicative of the Ni^2+^ in NiO and/or Ni(OH)_2_, while the second, at 857.8 eV, is associated with Ni^3+^ species. The presence of Ni^3+^ may indicate interactions between Ni and the ceria-based support, leading to partial oxidation of Ni. No clear Pt 4f, Ni 2p, or La 3d signals were observed for the PN_CZL_CNT catalyst, which suggests that either these metals were present in very low concentrations or were located beneath the oxide layer. The absence of La also suggests that it could be incorporated in the ceria–zirconia lattice to form CeZrLaO_2_ (confirmed by TEM-SAED; Figure S5), instead of forming separate La_2_O_3_ phases. Regarding content of lanthanum in PN_CZL_CNT catalyst determined by SEM/EDS (Table S2) and XPS (Table S4), the discrepancy in the results can be attributed to the different probing depths of the applied techniques. While EDS confirms the presence of La in the bulk of the catalyst (ca. 2 wt. %), and SAED indicates the formation of a CeZrLaO_2_ phase, XPS (which is a surface-sensitive technique) shows only a very weak La signal. This suggests that La is primarily incorporated into the CeZrO_2_ lattice or located beneath the surface rather than being enriched at the outermost surface layer. The results of XPS analyses suggest that the surface of PN_CZL_CNT is dominated by CeZrO_2_ species, which may favor CO_2_ activation but could limit methane activation due to reduced availability of exposed metal active sites.

### 2.2. Catalytic Tests

**Figure 8:**
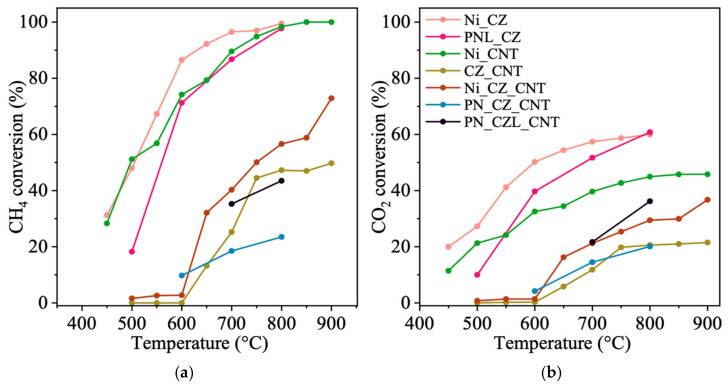
CH_4_ (**a**) and CO_2_ (**b**) conversions during DRM for CZ- and CNT-supported catalysts; reaction conditions: T = 500–900 °C, GHSV = 10,000 1/h, and CH_4_:CO_2_ = 1:2.5.

The performance of CNT-supported catalysts obtained in this work for methane dry reforming was evaluated under isothermal conditions and compared with that of two CeZrO_2_-supported catalysts (Ni_CZ and PNL_CZ) and two additional CNT-supported catalysts (Ni_CNT and Ni_CZ_CNT) described in our previous studies [42,50,51]. Figure 8 presents the conversions of CH_4_ and CO_2_ over the temperature range of 500–900 °C. As expected, due to the highly endothermic nature of DRM, the conversions of both reagents increase with increasing temperature. The higher CH_4_ conversion observed for monometallic Ni catalysts (Ni_CZ and Ni_CNT) is consistent with the presence of well-dispersed Ni nanoparticles, as evidenced by TEM analysis [42,50], which provide active sites for methane activation. In contrast, the incorporation of Pt and La results in a decrease in CH_4_ conversion, indicating that the introduction of these additional components does not enhance methane activation under the studied conditions. Although Ni_CZ displays the highest CH_4_ conversion, the addition of Pt was intended to modify catalyst stability and resistance to deactivation rather than maximize activity. The observed decrease indicated that in the present system, Pt alters the reaction pathway, favoring RWGS rather than enhancing CH_4_ activation. Moreover, the observed increase in CeZrO_2_ or CeZrLaO_2_ crystallite size (XRD, Table 1) and a decrease in specific surface area (S_BET_, Table 1) may indicate partial sintering and/or coverage of active Ni sites, which may limit CH_4_ activation. The observed decrease in CH_4_ conversion can also be associated with modifications of the Ni active phase. Interactions between Ni- and La-containing oxide species, as well as Ni-Pt interactions, may influence the properties of Ni sites responsible for methane activation. Furthermore, TEM and EDS analyses indicate close spatial proximity between metal and oxide phases, suggesting the presence of interfacial contact that may affect catalytic behavior. Such spatial distribution may lead to modification of Ni active sites, thereby reducing their intrinsic activity toward CH_4_ activation. Overall, the results suggest that preserving the intrinsic activity of Ni sites is more critical for DRM performance than the introduction of additional promoter phases.

Figure 8b displays higher CO_2_ conversions for CZ-supported catalysts, which can be attributed to the redox properties of Ce and CO_2_ adsorption occurring over oxygen vacancies [52]. Nevertheless, higher CO_2_ conversion does not necessarily correlate with higher DRM activity, as CO_2_ may also be consumed via the reverse water–gas shift (RWGS) reaction.

The CH_4_ conversions obtained in this study are comparable to those reported for Ni-based catalysts supported on ceria–zirconia systems under similar operating conditions [53]. However, upon the addition of Pt, a decrease in CH_4_ conversion is observed, indicating that Pt does not enhance methane activation in the present system. This suggests that the introduction of Pt modifies the local environment of Ni active sites, which may affect their catalytic properties. While several studies report that Pt promotes Ni dispersion and enhances DRM activity [8], other reports indicate that Pt may also facilitate hydrogen activation and thereby promote RWGS under specific conditions [54,55,56]. The present results are in agreement with the latter observations, suggesting that Pt can shift the reaction pathway toward hydrogen-consuming side reactions rather than improving methane activation. This suggests that the role of Pt is highly dependent on catalyst composition, metal loading, and preparation method, and may not universally lead to improved DRM performance.

**Figure 9:**
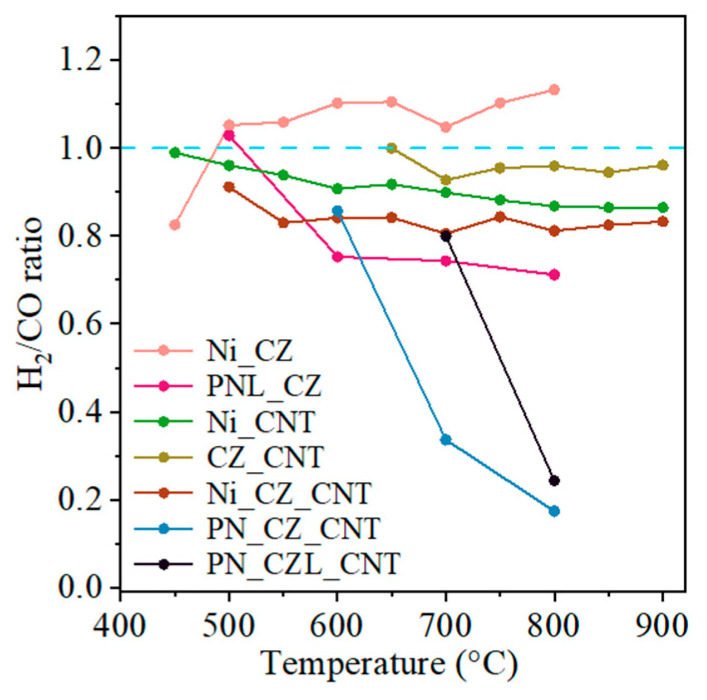
H_2_/CO ratio during DRM for CZ- and CNT-supported catalysts; reaction conditions: T = 500–900 °C, GHSV = 10,000 1/h, and CH_4_:CO_2_ = 1:2.5.

The H_2_/CO ratio is an important parameter for evaluating catalyst performance in DRM, as it reflects the relative contributions of the main reaction and competing side reactions. Under ideal DRM conditions, an H_2_/CO ratio close to unity is expected. As shown in Figure 9, most catalysts exhibit H_2_/CO ratios below 1, indicating that the produced H_2_ is partially consumed in parallel reactions, such as the reverse water–gas shift (RWGS) reaction [57]. A decrease in the H_2_/CO ratio due to competing RWGS reaction has been widely reported for Ni-based catalysts, particularly at elevated temperatures [58,59,60]. The trends observed in this work are consistent with these findings, although the extent of RWGS promotion appears to be more pronounced in Pt-containing systems. This highlights the importance of catalyst composition in determining the balance between DRM and RWGS pathways. A decrease in the H_2_/CO ratio is observed for Pt-containing catalysts, suggesting enhanced hydrogen consumption in these systems. This behavior may be associated with the ability of Pt to facilitate H_2_ activation, increasing the availability of reactive hydrogen species that participate in RWGS rather than DRM. This observation contrasts with literature reports where Pt addition improves Ni dispersion and enhances catalytic performance [56]. Additionally, the H_2_/CO ratio for Pt-containing catalysts decreases with increasing temperature, indicating that RWGS becomes more competitive under the studied conditions, particularly at elevated temperatures and in the presence of excess CO_2_. These results highlight that Pt-containing catalysts favor RWGS over DRM under CO_2_-rich conditions.

The H_2_/CO ratio for the Ni_CZ catalyst is slightly above 1 over the entire temperature range, which can be attributed to higher CH_4_ conversion and lower CO_2_ conversion. This suggests that methane decomposition contributes to H_2_ formation, potentially accompanied by carbon deposition, which may lead to catalyst deactivation. Such behavior may result from competing reactions of methane decomposition and the Boudouard reaction. In contrast, the remaining catalyst systems exhibit H_2_/CO ratios slightly below 1, reflecting higher CO_2_ conversion and lower H_2_ production. Among these, Ni_CZ_CNT shows the lowest H_2_/CO ratio (ca. 0.8 over the entire temperature range). For CNT-supported catalysts, a pronounced decrease in the H_2_/CO ratio is observed at around 800 °C. The extent of the RWGS reaction appears to be lower for La-containing catalysts (PN_CZL_CNT and PNL_CZ) compared to PN_CZ_CNT, as evidenced by a less significant decrease in the H_2_/CO ratio. This suggests that the presence of La may partially suppress the RWGS reaction.

SAED analysis indicates the formation of a Ni-Pt alloy, which has previously been reported to modify the electronic properties of Ni, improve metal dispersion, and reduce carbon deposition [18]. However, as evidenced by the H_2_/CO ratios for Pt-containing catalysts, alloy formation alone is not sufficient to suppress competing reactions, such as RWGS. Therefore, in these systems, the effect of alloying must be considered alongside other factors, including feed composition, the nature of the mixed oxide support, and interactions between the metal phases and the support. The mixed oxide phases (CeZrO_2_ and CeZrLaO_2_) also play an important role in catalyst stability due to their oxygen storage capacity and redox properties. These materials provide mobile lattice oxygen and oxygen vacancies, which can promote the oxidation of carbon intermediates formed during CH_4_ activation, thereby reducing carbon deposition. While they are unlikely to directly enhance methane activation, they contribute to CO_2_ activation and overall catalyst durability. Thus, the role of the mixed oxides is primarily associated with improved CO_2_ dissociative adsorption and support-assisted redox functionality rather than direct participation in methane reforming [57].

The results further show that the addition of La leads to a decrease in catalytic activity, as reflected by lower CH_4_ and CO_2_ conversions. Although La-containing oxides are known to enhance CO_2_ adsorption through increased basicity, this effect does not translate into improved DRM performance under the studied conditions. This may be attributed to interactions between La-containing species and the active metal phase (Ni). The resulting modifications in the local environment of Ni may affect the accessibility and/or reactivity of active sites involved in methane activation [61]. Overall, the incorporation of La appears to have a negative impact on DRM activity in the investigated systems.

**Figure 10:**
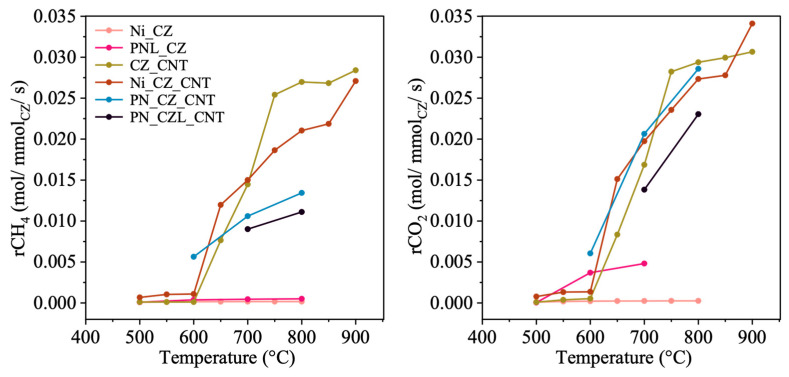
Conversion rates of CH_4_ and CO_2_ for CZ- and CNT-supported catalysts, normalized per mmol of CeZrO_2_ (CZ).

**Figure 11:**
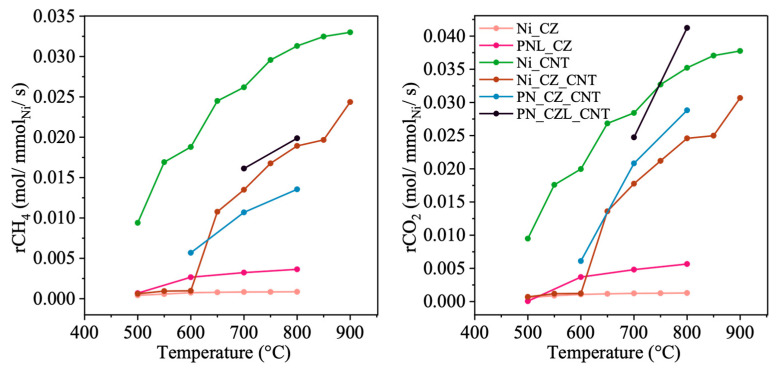
Conversion rates of CH_4_ and CO_2_ for CZ- and CNT-supported catalysts, normalized per mmol of Ni.

**Figure 12:**
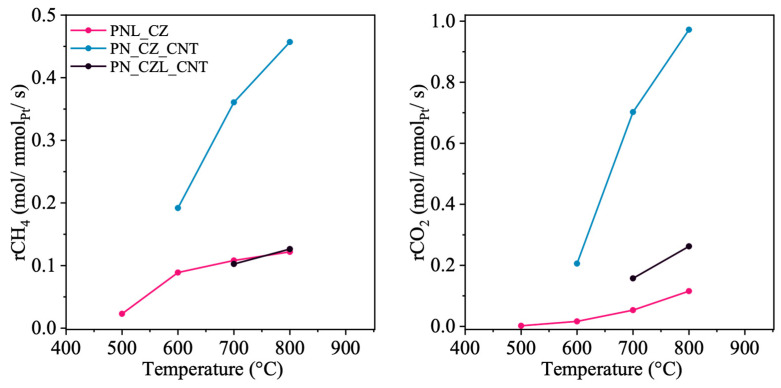
Conversion rates of CH_4_ and CO_2_ for CZ- and CNT-supported catalysts, normalized per mmol of Pt.

Figure 10, Figure 11 and Figure 12 illustrate the normalized conversion rates of CH_4_ and CO_2_ per mmol of the selected catalyst component, namely CeZrO_2_, CeZrLaO_2_, Ni, and Pt. These data are used to compare the relative contributions of individual components to the observed catalytic trends. The conversion of CH_4_ and CO_2_, as well as the corresponding normalized rates per mmol of active components, were calculated according to Equations (7)–(9).

When normalized per mmol of CeZrO_2_ (CZ), the conversion rates of both CH_4_ and CO_2_ are significantly lower for CZ-supported catalysts compared to CNT-supported systems. This can be attributed to the improved dispersion of active phases on CNTs, as evidenced by TEM analysis, which revealed finely distributed Ni nanoparticles on the CNT surface (Figures S2, S4 and S5, and our previous study [42]). Such dispersion facilitates better contact between reactants and active sites. Furthermore, the absence of distinct NiO reflections in the XRD patterns (Figure 1) suggests that large NiO crystallites were not formed. These structural features may contribute to the higher catalytic activity normalized to the Ni content observed for CNT-supported catalysts. As shown in Figure 10, the normalized conversion rates of CH_4_ and CO_2_ per mmol of CZ are highest for the CZ_CNT catalyst compared to Ni_CZ_CNT, despite the latter typically exhibiting higher overall catalytic activity in DRM. This behavior suggests a reduced contribution of the CZ phase to DRM in the presence of Ni, where methane activation is primarily governed by Ni sites. For PN_CZ_CNT and PN_CZL_CNT, the conversion rates normalized to CZ are further decreased, indicating that the relative contribution of the oxide phase to the overall reaction becomes less significant upon incorporation of additional metal components.

The rates of CH_4_ conversion over the active sites of Ni in CNT-supported catalysts show the highest activity for the Ni_CNT catalyst, supporting the dominant role of Ni in the activation of methane. The decrease in rates on the addition of other phases (Pt or La) suggests the modification of the local environment of Ni, which could have resulted in a change in Ni’s adsorption properties, possibly stronger interfacial interactions, or loss of accessibility of the sites. However, it is also seen that Pt with Ce, Zr, and La oxides improves the conversion rate of CO_2_ activation over Ni. The addition of La as a promoter increases the rate of conversion for both CH_4_ and CO_2_ over Ni with an increase in temperature, as a result of the endothermic nature of the reaction. The conversion for CO_2_ being comparatively higher than that of CH_4_ indicates additional CO_2_-consuming pathways, such as the occurrence of RWGS (Figure 11).

Figure 12 illustrates the normalized conversion rates of reactants over Pt active sites, which are found to depend on the catalyst composition. These results indicate that Pt is not the primary contributor to methane activation in the investigated systems. It is observed that the presence of La_2_O_3_ in the PN_CZL_CNT and PNL_CZ catalysts leads to a significant decrease in the conversion rates of both CH_4_ and CO_2_, which may be attributed to reduced accessibility of the active sites.

**Figure 13:**
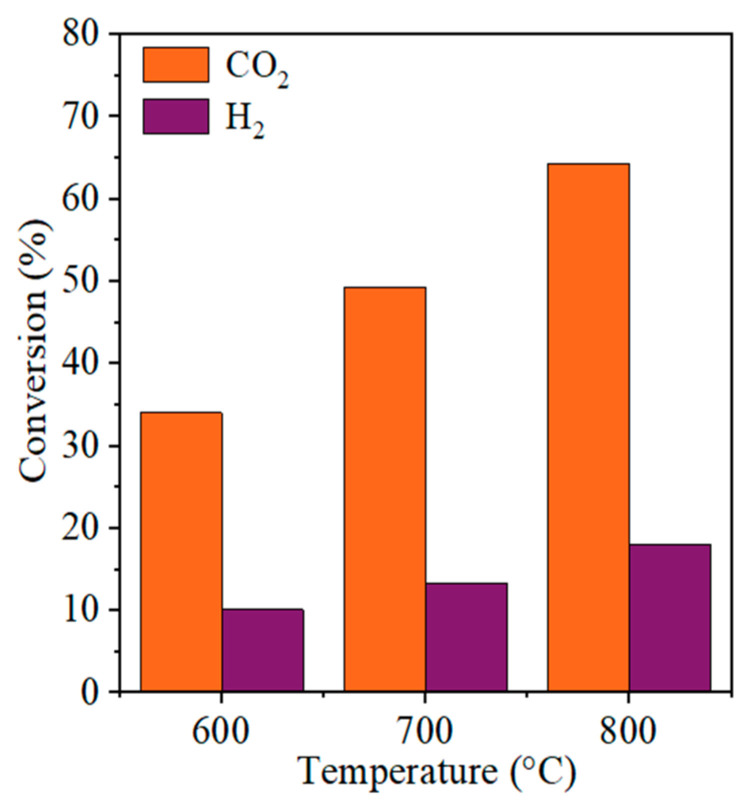
Conversion of CO_2_ and H_2_ during the catalytic test of RWGS over the PN_CZ_CNT catalyst in the temperature range of 600–800 °C.

To confirm the contribution of the RWGS reaction during DRM, an additional catalytic test was performed using the PN_CZ_CNT catalyst over the temperature range of 600–800 °C. Figure 13 presents the conversions of CO_2_ and H_2_ obtained during this test, both of which increase with increasing temperature, confirming that the catalyst is active in the RWGS reaction. This also explains the significant decrease in the H_2_/CO ratio over the catalyst during DRM tests. Although the RWGS test was conducted under H_2_-rich conditions, it provides clear evidence of the catalyst’s intrinsic ability to promote CO_2_ hydrogenation. This suggests that, during DRM, a fraction of the produced hydrogen is consumed via RWGS, thereby lowering the apparent efficiency of syngas production through DRM. Furthermore, the pronounced RWGS activity observed for the PN_CZ_CNT catalyst may be associated with the presence of Pt, which is known to facilitate hydrogen activation. The availability of reactive hydrogen species can enhance the RWGS pathway, especially at elevated temperatures, where thermodynamic conditions favor this reaction.

The DRM reaction over the investigated catalysts likely proceeds via a bifunctional pathway involving distinct roles of the metal and oxide phases. Ni acts as the primary active site for CH_4_ activation through dissociative adsorption, generating surface carbonaceous species and hydrogen. In parallel, CO_2_ is activated on the CeZrO_2_ or CeZrLaO_2_ mixed oxide phase, where oxygen vacancies and redox-active cerium sites facilitate its adsorption and dissociation. The resulting oxygen species can react with carbon-containing intermediates formed on Ni, thereby contributing to carbon removal and catalyst stability. The close spatial proximity between metal and oxide phases observed by TEM/EDS suggests that these interfacial regions may be important for coupling CH_4_ activation with CO_2_-derived oxygen transfer. In Pt-containing catalysts, Pt does not appear to be the main site for methane activation; instead, it may modify the local environment of Ni and promote hydrogen-mediated side reactions, particularly RWGS, as indicated by the decreased H_2_/CO ratios. Overall, the catalytic behavior is governed by the balance between Ni-driven CH_4_ activation, oxide-assisted CO_2_ activation, and competing hydrogen-consuming pathways.

## 3. Materials and Methods

### 3.1. Materials

Multi-walled carbon nanotubes (CNTs, outer diameter of 50–90 nm, length of >6.5 μm, and >95% carbon), cerium(III) nitrate hexahydrate (Ce(NO_3_)_3_⋅6H_2_O, 99%), and zirconium(IV) oxynitrate hydrate (N_2_O_7_Zr⋅H_2_O, 99%) were purchased from Sigma-Aldrich (St. Louis, MO, USA). Lanthanum(III) acetate sesquihydrate (La(CH_3_COO)_3_⋅1.5H_2_O, 99.9%) was purchased from Thermo Fisher GmbH (Dreieich, Germany). Bis(acetylacetonato) platinum(II) (Pt(O_2_C_5_H_7_)_2_, 97%) and nickel(II) bis(acetylacetonate) (Ni(acac)_2_) were purchased from Angene International (Nanjing, China). Benzoic acid (C_6_H_5_COOH, 99.5%) was purchased from Chempur (Piekary Śląskie, Poland). Solvents, i.e., ethyl alcohol (C_2_H_5_OH, 96%), acetone ((CH_3_)_2_CO, ACS grade), nitric acid (HNO_3_, 68%), and dimethylformamide ((CH_3_)_2_NCH, 99.8%), were purchased from STANLAB (Lublin, Poland). All reagents were used without further purification. Water was distilled using the Hydrolab Distiller (Hydrolab, Straszyn, Poland).

### 3.2. Catalyst Synthesis

Carbon nanotubes (CNTs) were used as support to synthesize three catalysts (CZL_CNT, PN_CZ_CNT, and PN_CZL_CNT). Prior to synthesis, CNTs (1 g) were treated with 400 mL of concentrated (68%) HNO_3_ at 120 °C for 24 h to introduce surface functional groups. After this treatment, CNTs were repeatedly washed with distilled water until the filtrate reached neutral pH and subsequently dried at 120 °C for 24 h. The catalysts were synthesized using a sequential deposition approach in which Ce-Zr or Ce-Zr-La mixed oxides were first deposited onto the functionalized CNTs, followed by incorporation of Ni and Pt active phases.

For the synthesis of CZ_CNT catalyst, 100 mg of functionalized CNTs were dispersed in 40 mL of ethanol and 50 mL of distilled water, and suspended in a round-bottom flask equipped with a water condenser. Cerium and zirconium salts (47.1 mg and 11.3 mg, respectively) were dissolved in 50 mL of a mixture of distilled water and acetone (1:1, *v*/*v*) and added dropwise to the CNT suspension. The mixture was sonicated for 1 h. To provide an inert environment, the suspension was continuously purged with Ar at a flow rate of 10 mL/min. After 1 h, the pH of the suspension was adjusted to 10 using 1 M NaOH solution. The suspension was then stirred at 70 °C for 4 h and subsequently at room temperature overnight. The resulting product (CZ_CNT) was filtered off, washed with water until it had a neutral pH, and dried at 100 °C for 24 h.

For the synthesis of the CZL_CNT catalyst, in a round-bottom flask, 100 mg of functionalized CNTs were suspended in 40 mL of ethanol and 50 mL of distilled water. The flask was connected to a water condenser. Cerium, zirconium, and lanthanum salts (148 mg, 57.1 mg, and 10.1 mg, respectively) were dissolved in approximately 40 mL of a mixture of distilled water and ethanol (1:1, *v*/*v*) and added dropwise to the CNT suspension. The mixture was sonicated for 1 h. To provide an inert environment, the suspension was continuously purged with Ar at a flow rate of 10 mL/min. After 1 h, the pH of the suspension was adjusted to 10 using 1 M NaOH solution. The suspension was then stirred at 70 °C for 4 h and subsequently at room temperature overnight. The resulting product (CZL_CNT) was filtered, washed with water until it had a neutral pH, and dried at 100 °C for 24 h.

Synthesis of PN_CZ_CNT catalyst. The CZ_CNT was re-dispersed in a 50 mL mixture of acetone and distilled water (1:1, *v*/*v*) and sonicated for 1 h. Nickel and platinum salts (41.9 mg and 1.9 mg, respectively) were dissolved separately in 10 mL and 20 mL of acetone–water mixtures (1:1 and 1:3, *v*/*v*, respectively) and added dropwise to the CZ_CNT suspension. The mixture was sonicated, followed by the addition of NaOH until the pH reached 10. It was then heated at 70 °C for 4 h and stirred overnight at room temperature. The resulting PN_CZ_CNT catalyst was filtered-off, washed with distilled water until a neutral pH was achieved, and subsequently dried at 100 °C overnight.

Synthesis of PN_CZL_CNT catalyst. CZL_CNT was suspended in 111 mL of DMF and sonicated for 30 min, followed by the addition of 76.9 mg and 18.5 mg of nickel and platinum salts, respectively, along with 0.456 g of benzoic acid. The mixture was sonicated again and then transferred to a Teflon-lined steel reactor, in which it was heated at 160 °C for 24 h. The obtained PN_CZL_CNT catalyst was centrifuged, washed until the pH was neutral, and vacuum-dried overnight at 130 °C.

The nominal loadings of metals deposited on CNTs are provided in Table S1, which were calculated from the initial amounts of precursor salts used during synthesis, assuming complete incorporation of metal species into the catalyst. The composition of catalysts determined by EDS and XPS is provided in Tables S2–S4.

### 3.3. Catalyst Characterization

X-ray diffraction (XRD) patterns were recorded over a 2θ range from 10 to 80° with a step size of 0.03° using the MiniFlex diffractometer (Rigaku, Tokyo, Japan) equipped with Cu Kα radiation (λ = 1.54 Å). The specific surface area of the catalyst samples was determined by N_2_ adsorption–desorption at 77 K using an Autosorb-1 apparatus (Quantachrome Instruments, Boynton Beach, FL, USA). The surface area was calculated using the Brunauer–Emmett–Teller (BET) method, while the pore volume was determined using the Barrett–Joyner–Halenda (BJH) method. Prior to analysis, the samples were degassed at 150 °C for 12 h. The morphology of the obtained catalysts was examined by transmission electron microscopy using the S/TEM Titan 80–300 microscope (FEI company, Hillsboro, OR, USA) equipped with an EDAX energy-dispersive X-ray spectroscopy (EDS) detector, operated at an accelerating voltage of 300 kV. XPS analyses were carried out in a PHI VersaProbeII Scanning XPS system (Physical Electronics, Chanhassen, MN, USA) using monochromatic Al Kα (1486.6 eV) X-rays focused to a 100 µm spot and scanned over an area of 400 µm × 400 µm. The photoelectron take-off angle was 45°, and the pass energy in the analyzer was set to 46.95 eV (0.1 eV step) to obtain high-energy resolution spectra for the C 1s, O 1s, Zr 3d, Ce 3d, Ni 2p, La 3d, and Pt 4f regions. A dual-beam charge compensation with 7 eV Ar^+^ ions and 1 eV electrons was used to maintain a constant sample surface potential regardless of the sample conductivity. All XPS spectra were charge-referenced to the saturated carbon (C-C) C 1s peak at 285.0 eV. The operating pressure in the analytical chamber was less than 3 × 10^−9^ mbar. Deconvolution of spectra was carried out using PHI MultiPak software (v.9.9.3). Spectrum background was subtracted using the Shirley method. Within the experiment geometry, the information depth of analysis was about 5 nm.

### 3.4. Catalytic Tests

The performance of all catalysts in DRM and RWGS reactions was evaluated under isothermal conditions. The experiments were carried out in a U-shaped fixed-bed reactor placed in a temperature-controlled oven. All tests were performed at atmospheric pressure.

The DRM catalytic tests were conducted using a reaction mixture composed of 4 vol. % CH_4_, 10 vol. % CO_2_, and balance Ar (CH_4_:CO_2_ = 1:2.5.). The gas hourly space velocity (GHSV) was maintained at 10,000 1/h for all experiments. Isothermal tests were conducted over the temperature range of 500–900 °C. The initial temperature was set at 900 °C and subsequently decreased in 50 °C intervals after 2 h on stream. The same catalyst sample was used throughout the temperature sequence.

The reaction products were analyzed using a AI 93 gas chromatograph (AI, Cambridge, UK) equipped with a thermal conductivity detector (TCD) and two packed columns for the separation of H_2_, CH_4_, CO, and CO_2_. A molecular sieve-packed column was used for the separation of H_2_ and CO, while a Porapak Q-packed column (Agilent, Santa Clara, CA, USA) enabled the detection of CH_4_ and CO_2_. The TCD signal was integrated and processed using the SCL-10A VP system controller (Shimadzu, Kyoto, Japan). The GC was calibrated using a gas mixture containing 10 vol. % of CH_4_, 10 vol. % of CO_2_, 10 vol. % of H_2,_ and 10 vol. % of CO, balanced with Ar. The carrier gas was Ar. Calibration curves were established by correlating the detected peak areas with the molar concentration of each component. The molar fractions of the outlet gases were calculated from the measured peak areas using the corresponding calibration constants. The outlet molar flow rates of the gas components were determined based on the inlet flow rate and the measured outlet composition, assuming ideal gas behavior and a constant flow rate.

The conversions of CH_4_ and CO_2_, as well as the normalized rates of CH_4_ and CO_2_ conversion per mmol of the active components, were calculated based on the equations listed as follows:

(7) $$X_{i}=\frac{F_{i_{\;in}}-\;F_{i_{\;out}}}{F_{i_{\;in}}}\;\cdot 100$$

(8) $$r_{i}=\frac{F_{i_{\;in}}-F_{i_{\;out}}}{n_{a}}$$

(9) $$x_{a}=\frac{n_{a}}{n_{tot}}\;$$

where:

*X_i_*—conversion of component *i* (CH_4_ or CO_2_) (%);
*F_i_*—molar flow rate of component *i* (CH_4_ or CO_2_) (mol/s);
*r_i_*—rate of conversion of component *i* (mol/mmol/s);
*n_a_*—amount of the selected active component (CeZrO_2_, CeZrLaO_2_, Ni, or Pt) (mmol);
*n_tot_*—total amount of active components present in the catalyst (CeZrO_2_, CeZrLaO_2_, Ni, or Pt) (mmol);
*x_a_*—molar fraction of a given active component in the catalyst.

The reported values correspond to apparent conversion rates normalized to the amount of catalyst component, which is used for comparative analysis of catalytic performance. These values do not represent intrinsic kinetic rates because detailed kinetic measurements under differential conditions were not performed. To evaluate the intrinsic activity of the catalyst in the reverse water–gas shift (RWGS) reaction, additional tests were carried out using a reaction mixture composed of 4 vol. % CO_2_, 12 vol. % H_2_, and Ar as a balance (CO_2_:H_2_ = 1:3). Excess H_2_ was used to ensure that hydrogen availability did not limit CO_2_ conversion, thereby enabling a clear assessment of RWGS activity. The gas hourly space velocity (GHSV) was maintained at 10,000 1/h for all experiments. Isothermal tests were conducted over the temperature range of 600–800 °C. The initial temperature was set at 800 °C and subsequently decreased in 100 °C intervals after 2 h on stream. The same catalyst sample was used throughout the temperature sequence.

## 4. Conclusions

In this study, CNT-supported catalysts incorporating Pt and Ni on CeZrO_2_ and CeZrLaO_2_ were synthesized and tested for dry reforming of methane (DRM) under CO_2_-rich conditions. Characterization by XRD, N_2_ sorption, SEM, TEM, SAED, and XPS confirmed the successful formation of CeZrO_2_ and CeZrLaO_2_ mixed oxides on the CNT surface, with uniform dispersion of the active phases. The synthesis process also led to co-dispersion of Pt and Ni over the mixed oxides and the CNTs.

The CNT-supported catalysts exhibited significant activity in DRM, with higher CH_4_ and CO_2_ conversion rates compared to catalysts supported only on ceria–zirconia. This enhanced performance is attributed to improved contact between the CNT support and metal active sites. The presence of Pt-Ni in PN_CZ_CNT and PN_CZL_CNT catalysts was found to promote the reverse water–gas shift (RWGS) reaction, consuming both CO_2_ and H_2_. The addition of Pt to the Ni-based catalysts led to a reduction in DRM activity compared to Pt-free counterparts. Furthermore, the introduction of La resulted in decreased CH_4_ and CO_2_ conversion rates. Given the known role of La-containing oxides in mitigating carbon deposition, further investigation into the role of La in CNT-supported bimetallic catalysts is needed to clarify its impact on DRM activity. Overall, despite the formation of a Pt-Ni alloy, these catalysts demonstrated stronger activity toward the RWGS reaction than DRM, warranting further optimization.

## Figures and Tables

**Table 1:** The average crystallite size (D) of CeZrO_2_ or CeZrLaO_2_ determined from the (111), (200), (202), and (311) reflections using the Scherrer Equation, and the textural properties of CNTs and catalysts (S_BET_—surface area determined using the Brunauer–Emmett–Teller (BET) method, V_t_—total pore volume, and d—average pore size).

Sample	D_111_ (nm)	D_200_ (nm)	D_220_ (nm)	D_311_ (nm)	S_BET_ (m^2^/g)	V_t_ (cm^3^/g)	d (nm)
CNT	-	-	-	-	58	0.22	13.20
CZL_CNT	3.41	8.82	3.16	3.05	176	0.24	5.57
PN_CZ_CNT	4.98	5.51	3.49	2.62	159	0.26	6.61
PN_CZL_CNT	6.03	6.92	4.82	5.70	150	0.41	10.43

## Data Availability

The data presented in this study are available on request from the corresponding author.

## Supplementary Materials

The following supporting information can be downloaded at: https://www.mdpi.com/article/10.3390/molecules31101655/s1; Figure S1. SEM images of (a) CNT, (b) CZL_CNT, (c) PN_CZ_CNT, and (d) PN_CZL_CNT; Figure S2. Dark field TEM images of (a) CZL_CNT, (b) PN_CZ_CNT, and (c) PN_CZL_CNT; Figure S3. (a) HRTEM image of the catalyst CZL_CNT showing the lattice structure of the catalyst. (b) DF-TEM highlighting crystalline nanoparticles dispersed on the CNTs, circled region indicating area selected for SAED, and (c) SAED pattern of the circled region showing diffraction rings indexed to the CeZrLaO_2_ support; Figure S4. (a) HRTEM image of the catalyst PN_CZ_CNT showing the lattice structure of the catalyst; (b) DF-TEM highlighting crystalline nanoparticles dispersed on the CNTs, with the circled region indicating the area selected for SAED; (c) SAED pattern of the circled region showing diffraction rings indexed to the CeZrO_2_ support; and (d) SAED pattern of the circled region showing diffraction rings attributed to Ni-Pt containing phases; Figure S5. (a) HRTEM image of the catalyst PN_CZL_CNT showing the lattice structure of the catalyst; (b) DF-TEM highlighting crystalline nanoparticles dispersed on the CNTs, with the circled region indicating the area selected for SAED; (c) SAED pattern of the circled region showing diffraction rings indexed to the CeZrLaO_2_ support; and (d) SAED pattern of the circled region showing diffraction rings attributed to Ni-Pt containing phases; Figure S6. XPS survey spectra of (a) PN_CZ_CNT and (b) PN_CZL_CNT catalysts; Table S1. Nominal loading of metals in obtained CNT-supported catalysts; Table S2. Average metal loading in CNT-supported catalysts determined by energy-dispersive X-ray spectroscopy (EDS); Table S3. Surface composition (atomic %) of C and O in catalysts determined by XPS; Table S4. Surface composition (atomic %) of metals in catalysts determined by XPS; Table S5. Carbon balance calculated from Equation (S1) during catalytic tests of DRM (4 vol. % CH_4_, 10 vol. % CO_2_, balanced with Ar; TOS = 2 h, GHSV = 10,000 1/h); Table S6. Hydrogen balance calculated from Equation (S2) during catalytic tests of DRM (4 vol. % CH_4_, 10 vol. % CO_2_, balanced with Ar; TOS = 2 h, GHSV = 10,000 1/h).

## References

[1] Farooqi A.S., Yusuf M., Mohd Zabidi N.A., Saidur R., Sanaullah K., Farooqi A.S., Khan A., Abdullah B. (2021). A Comprehensive Review on Improving the Production of Rich-Hydrogen via Combined Steam and CO_2_ Reforming of Methane over Ni-Based Catalysts. Int. J. Hydrogen Energy.

[2] Guharoy U., Reina T.R., Liu J., Sun Q., Gu S., Cai Q. (2021). A Theoretical Overview on the Prevention of Coking in Dry Reforming of Methane Using Non-Precious Transition Metal Catalysts. J. CO2 Util..

[3] Alawi N.M., Al-Mohammedawi H.H., Nguyen H.M., Azeez R.A., Shams O.A., Sukkar K.A. (2024). Catalysts for Reforming of Methane (A Review). Pet. Chem..

[4] Hussien A.G.S., Polychronopoulou K. (2022). A Review on the Different Aspects and Challenges of the Dry Reforming of Methane (DRM) Reaction. Nanomaterials.

[5] le Saché E., Reina T.R. (2022). Analysis of Dry Reforming as Direct Route for Gas Phase CO_2_ Conversion. The Past, the Present and Future of Catalytic DRM Technologies. Prog. Energy Combust. Sci..

[6] Bhaskaran A., Roy S. (2025). Exploring Dry Reforming of CH4 to Syngas Using High-Entropy Materials: A Novel Emerging Approach. ChemCatChem.

[7] Nguyen D.L.T., Vy Tran A., Vo D.-V.N., Tran Nguyen H., Rajamohan N., Trinh T.H., Nguyen T.L., Le Q.V., Nguyen T.M. (2024). Methane Dry Reforming: A Catalyst Challenge Awaits. J. Ind. Eng. Chem..

[8] Ranjekar A.M., Yadav G.D. (2021). Dry Reforming of Methane for Syngas Production: A Review and Assessment of Catalyst Development and Efficacy. J. Indian Chem. Soc..

[9] Zhu H., Chen H., Zhang M., Liang C., Duan L. (2024). Recent Advances in Promoting Dry Reforming of Methane Using Nickel-Based Catalysts. Catal. Sci. Technol..

[10] Sharifianjazi F., Esmaeilkhanian A., Bazli L., Eskandarinezhad S., Khaksar S., Shafiee P., Yusuf M., Abdullah B., Salahshour P., Sadeghi F. (2022). A Review on Recent Advances in Dry Reforming of Methane over Ni- and Co-Based Nanocatalysts. Int. J. Hydrogen Energy.

[11] Xin J., Cui H., Cheng Z., Zhou Z. (2018). Bimetallic Ni-Co/SBA-15 Catalysts Prepared by Urea Co-Precipitation for Dry Reforming of Methane. Appl. Catal. A Gen..

[12] Khani Y., Pyo S., Bahadoran F., Cho K., Jeong K.-E., Park Y.-K. (2025). Synthesis of Coke-Resistant Catalyst Using NiAl_2_O_4_ Support for Hydrogen Production via Autothermal Dry Reforming of Methane. ChemCatChem.

[13] Küchen G., Olszok V., Kreitz B., Mahr C., Rosenauer A., Turek T., Weber A.P., Wehinger G.D. (2024). Spray-Dried Ni-Co Bimetallic Catalysts for Dry Reforming of Methane. ChemCatChem.

[14] Dhillon G.S., Cao G., Yi N. (2023). The Role of Fe in Ni-Fe/TiO_2_ Catalysts for the Dry Reforming of Methane. Catalysts.

[15] Kong L., Qin L., Zhao B., Yang Q., Han J. (2024). Preparation of Nanoscale Ni–Cu Supported Over Hydrochar by Hydrothermal Method and Effect of Ni/Cu Ratio on Catalytic Performances in Dry Reforming of Methane. Catal. Lett..

[16] Shi C., Wang S., Ge X., Deng S., Chen B., Shen J. (2021). A Review of Different Catalytic Systems for Dry Reforming of Methane: Conventional Catalysis-Alone and Plasma-Catalytic System. J. CO2 Util..

[17] Araiza D.G., Arcos D.G., Gómez-Cortés A., Díaz G. (2021). Dry Reforming of Methane over Pt-Ni/CeO_2_ Catalysts: Effect of the Metal Composition on the Stability. Catal. Today.

[18] Niu J., Wang Y., Liland S.E., Regli S.K., Yang J., Rout K.R., Luo J., Rønning M., Ran J., Chen D. (2021). Unraveling Enhanced Activity, Selectivity, and Coke Resistance of Pt–Ni Bimetallic Clusters in Dry Reforming. ACS Catal..

[19] Gao X., Ge Z., Zhu G., Wang Z., Ashok J., Kawi S. (2021). Anti-Coking and Anti-Sintering Ni/Al_2_O_3_ Catalysts in the Dry Reforming of Methane: Recent Progress and Prospects. Catalysts.

[20] Ma X., Yang W.-W., Zhang J.-R., Tang X.-Y. (2025). Structural Evolution of Ni-Ce Bimetallic Alloy on Al_2_O_3_ Support in Methane Dry Reforming: Achieving Sustainability and High-Efficiency Reaction through Cerium Modulation Strategy. Fuel.

[21] Babakouhi R., Alavi S.M., Rezaei M., Jokar F., Varbar M., Akbari E. (2024). Hydrogen Production through Combined Dry Reforming and Partial Oxidation of Methane over the Ni/Al_2_O_3_–CeO_2_ Catalysts. Int. J. Hydrogen Energy.

[22] Liu Y., Jin H., Huang L., Liu Y., Cui S., Liu H., Zeng S., Wang L. (2024). Anti-Coking Ni-La_2_O_3_/SiO_2_ Catalyst Prepared by Using a Glycine-Assisted Impregnation Method for Low-Temperature Dry Reforming of Methane. Chem. Lett..

[23] Androulakis A., Yentekakis I.V., Panagiotopoulou P. (2023). Dry Reforming of Methane over Supported Rh and Ru Catalysts: Effect of the Support (Al_2_O_3_, TiO_2_, ZrO_2_, YSZ) on the Activity and Reaction Pathway. Int. J. Hydrogen Energy.

[24] Jin H., Liu Y., Huang L., Liu Y., Cui S., Liu H., Xu J., Wang L. (2024). Three-Dimensional Mesoporous Ni-CeO_2_ Catalyst for Dry Reforming of Methane. Catalysts.

[25] Tu P.H., Le D.N., Dao T.D., Tran Q.-T., Doan T.C.D., Shiratori Y., Dang C.M. (2020). Paper-Structured Catalyst Containing CeO_2_–Ni Flowers for Dry Reforming of Methane. Int. J. Hydrogen Energy.

[26] Wang Y., Li R., Zeng C., Sun W., Fan H., Ma Q., Zhao T.-S. (2025). Recent Research Progress of Methane Dry Reforming to Syngas. Fuel.

[27] Horváth A., Németh M., Beck A., Maróti B., Sáfrán G., Pantaleo G., Liotta L.F., Venezia A.M., La Parola V. (2021). Strong Impact of Indium Promoter on Ni/Al_2_O_3_ and Ni/CeO_2_-Al_2_O_3_ Catalysts Used in Dry Reforming of Methane. Appl. Catal. A Gen..

[28] Zhang F., Gutiérrez R.A., Lustemberg P.G., Liu Z., Rui N., Wu T., Ramírez P.J., Xu W., Idriss H., Ganduglia-Pirovano M.V. (2021). Metal–Support Interactions and C1 Chemistry: Transforming Pt-CeO_2_ into a Highly Active and Stable Catalyst for the Conversion of Carbon Dioxide and Methane. ACS Catal..

[29] Zhou X., Gao Y., Yang J., Yi W., Pang Q., Liu Z., Liu B., Zhang M. (2023). Unraveling the Effects of Ce/Zr Molar Ratio in Mesoporous CexZr_1−x_O_2_ on the Performance of Dry Reforming of Methane over the Supported Ni Catalysts. Chem. Eng. Res. Des..

[30] Dekkar S. (2024). Dry Reforming of Methane Over Ni/ZrO_2_, Ni/CeO_2_ and Ni/La_2_O_3_ Catalysts: Role of Support Nature and Its Synthesis by Microemulsion Method. Chem. Afr..

[31] Zou Z., Zhang T., Lv L., Tang W., Zhang G., Gupta R.K., Wang Y., Tang S. (2023). Preparing a Zr-Doped CeO_2_ Nanorod to Improve the Catalytic Performance of the Ni-Based Catalyst for Dry Reforming of Methane by Enhancing Oxygen Supply. ACS Sustain. Chem. Eng..

[32] Phichairatanaphong O., Donphai W. (2023). Role of Cerium–Zirconium Ratio and Chemical Surface Property of CeO_2_–ZrO_2_ Supported Nickel-Based Catalysts in Dry Reforming Reaction. Top. Catal..

[33] Jagódka P., Matus K., Sobota M., Łamacz A. (2021). Dry Reforming of Methane over Carbon Fibre-Supported CeZrO_2_, Ni-CeZrO_2_, Pt-CeZrO_2_ and Pt-Ni-CeZrO_2_ Catalysts. Catalysts.

[34] Wang F., Xu L., Yang J., Zhang J., Zhang L., Li H., Zhao Y., Li H.X., Wu K., Xu G.Q. (2017). Enhanced Catalytic Performance of Ir Catalysts Supported on Ceria-Based Solid Solutions for Methane Dry Reforming Reaction. Catal. Today.

[35] Mesrar F., Kacimi M., Liotta L.F., Puleo F., Ziyad M. (2018). Syngas Production from Dry Reforming of Methane over Ni/Perlite Catalysts: Effect of Zirconia and Ceria Impregnation. Int. J. Hydrogen Energy.

[36] Xu Y., Qiao J., Sun W., Wang Z., Sun K. (2024). Enhancement of CO_2_ Activation and Coke-Resistant Ability on Ni/CeO_2_ Catalyst with La Doping for Dry Reforming of Methane. Int. J. Hydrogen Energy.

[37] Charisiou N.D., Tzounis L., Sebastian V., Hinder S.J., Baker M.A., Polychronopoulou K., Goula M.A. (2019). Investigating the Correlation between Deactivation and the Carbon Deposited on the Surface of Ni/Al_2_O_3_ and Ni/La_2_O_3_-Al_2_O_3_ Catalysts during the Biogas Reforming Reaction. Appl. Surf. Sci..

[38] Mierczynski P., Mosinska M., Stepinska N., Chalupka K., Nowosielska M., Maniukiewicz W., Rogowski J., Goswami N., Vasilev K., Szynkowska M.I. (2021). Effect of the Support Composition on Catalytic and Physicochemical Properties of Ni Catalysts in Oxy-Steam Reforming of Methane. Catal. Today.

[39] Li S., Fu Y., Kong W., Wang J., Yuan C., Pan B., Zhu H., Chen X., Zhang Y., Zhang J. (2023). Tuning Strong Metal-Support Interactions to Boost Activity and Stability of Aluminium Nitride Supported Nickel Catalysts for Dry Reforming of Methane. Fuel.

[40] You J., Lai L., Chen Y. (2025). Recent Advances in Strong Metal-Support Interaction Engineering for Dry Reforming of Methane Catalysts. Small.

[41] Alipour Z., Babu Borugadda V., Wang H., Dalai A.K. (2023). Syngas Production through Dry Reforming: A Review on Catalysts and Their Materials, Preparation Methods and Reactor Type. Chem. Eng. J..

[42] Łamacz A., Jagódka P., Stawowy M., Matus K. (2020). Dry Reforming of Methane over CNT-Supported CeZrO_2_, Ni and Ni-CeZrO_2_ Catalysts. Catalysts.

[43] Figueira C.E., Moreira P.F., Giudici R., Alves R.M.B., Schmal M. (2018). Nanoparticles of Ce, Sr, Co in and out the Multi-Walled Carbon Nanotubes Applied for Dry Reforming of Methane. Appl. Catal. A Gen..

[44] Lee G.-W., Kim J., Yoon J., Bae J.-S., Shin B.C., Kim I.S., Oh W., Ree M. (2008). Structural Characterization of Carboxylated Multi-Walled Carbon Nanotubes. Thin Solid Films.

[45] Kozonoe C.E., Santos V.M., Schmal M. (2023). Investigating the Stability of Ni and Fe Nanoparticle Distribution and the MWCNT Structure in the Dry Reforming of Methane. Environ. Sci. Pollut. Res..

[46] Oh S.H., Kim H.-K., Park S.-Y., Kim Y.-C., Kwon D.-H., Yang S., Ji H.-I., Chang H.J., Yoon K.J., Son J.-W. (2024). Investigating the Nano-Scale Structure and Composition Dynamics during the Phase Transition towards Complete Separation of CeO_2_–ZrO_2_ Solid Solutions. J. Mater. Chem. A.

[47] Muhich C.L. (2017). Re-Evaluating CeO_2_ Expansion Upon Reduction: Noncounterpoised Forces, Not Ionic Radius Effects, Are the Cause. J. Phys. Chem. C.

[48] Abidin S.Z., Mohamad I.S., Hashim A.Y.B., Abdullah N. (2018). Textural and Adsorption Analysis of Nanocarbon Particles. Int. J. Nanoelectron. Mater..

[49] Jagódka P., Matus K., Łamacz A. (2022). On the HKUST-1/GO and HKUST-1/rGO Composites: The Impact of Synthesis Method on Physicochemical Properties. Molecules.

[50] Łamacz A., Pawlyta M., Dobrzański L.A., Krztoń A. (2011). Characterization of the Structure Features of CeZrO_2_ and Ni/CeZrO_2_ Catalysts for Tar Gasification with Steam. Arch. Mater. Sci. Eng..

[51] Łamacz A., Matus K., Liszka B., Silvestre-Albero J., Lafjah M., Dintzer T., Janowska I. (2018). The Impact of Synthesis Method of CNT Supported CeZrO_2_ and Ni-CeZrO_2_ on Catalytic Activity in WGS Reaction. Catal. Today.

[52] Marinho A.L.A., Rabelo-Neto R.C., Bion N., Toniolo F.S., Noronha F.B. (2024). Dry Reforming of Methane over Embedded Ni Nanoparticles in CeZrO_2_: Effect of Ce/Zr Ratio and H_2_O Addition. Int. J. Hydrogen Energy.

[53] Sophiana I.C., Steven S., Shalihah R.K., Iskandar F., Devianto H., Restiawaty E., Nishiyama N., Budhi Y.W. (2024). Enhanced Syngas Production through Dry Reforming of Methane with Ni/CeZrO_2_ Catalyst: Kinetic Parameter Investigation and CO_2_-Rich Feed Simulation. Chem. Eng. J. Adv..

[54] Xie J., Feng Y., Wang X., Li X., Yu J., Gao A., Jiang J., Chang Q., Dai Y., Liu W. (2025). Fully Exposed Platinum Clusters for the Efficient Reverse Water-Gas Shift Reaction at Low Temperatures. Appl. Catal. B Environ. Energy.

[55] Zhou C., Zhang J., Fu Y., Dai H. (2023). Recent Advances in the Reverse Water–Gas Conversion Reaction. Molecules.

[56] Jawad A. (2023). The Effects of Fe, Mg, and Pt-Doping on the Improvement of Ni Stabilized on Al_2_O_3_-CeO_3_ Catalysts for Methane Dry Reforming. RSC Adv..

[57] Ighalo J.O., Paddock M.D., Almkhelfe H., Nepal A., Lacroix B., He X., Anthony J.L., Amama P.B. (2025). Dry Reforming of Methane at High Space Velocities on CeO_2_-Supported Ni Catalysts. Chem. Eng. J..

[58] Bach V.R., de Camargo A.C., de Souza T.L., Cardozo-Filho L., Alves H.J. (2020). Dry Reforming of Methane over Ni/MgO–Al_2_O_3_ Catalysts: Thermodynamic Equilibrium Analysis and Experimental Application. Int. J. Hydrogen Energy.

[59] Niu J., Guo F., Ran J., Qi W., Yang Z. (2020). Methane Dry (CO_2_) Reforming to Syngas (H_2_/CO) in Catalytic Process: From Experimental Study and DFT Calculations. Int. J. Hydrogen Energy.

[60] Wang Y., Yao L., Wang S., Mao D., Hu C. (2018). Low-Temperature Catalytic CO_2_ Dry Reforming of Methane on Ni-Based Catalysts: A Review. Fuel Process. Technol..

[61] Chen M., Wang L. (2024). Performance of Ni-Based Catalysts with La Promoter for the Reforming of Methane in Gasification Process. Catalysts.

