# Supplementary material for: CNT-Supported Pt-Ni Catalysts Promoted with CeZrO2 and CeZrLaO2 for Dry Reforming of Methane
Source: Molecules. 2026 May 14;31(10):1655. doi: 10.3390/molecules31101655 (PMC13210149; doi:10.3390/molecules31101655)
Supplement: Supplementary file 1 [file molecules-31-01655-s001.zip › molecules-4211308-supplementary.pdf]

Supplementary File

CNT-Supported Pt-Ni Catalysts Promoted with $CeZrO_2$ and $CeZrLaO_2$ for Dry Reforming of Methane

Mahima Kamra [1,*], Krzysztof Matus [2] and Agata Łamacz [1,*]

1. Department of Engineering and Technology of Chemical Processes, Wrocław University of Science and Technology, 50-370 Wrocław, Poland
2. Materials Research Laboratory, Faculty of Mechanical Engineering, Silesian University of Technology, 41-100 Gliwice, Poland; krzysztof.matus@polsl.pl

* Correspondence: mahima.kamra@pwr.edu.pl (M.K.); agata.lamacz@pwr.edu.pl (A.Ł.)

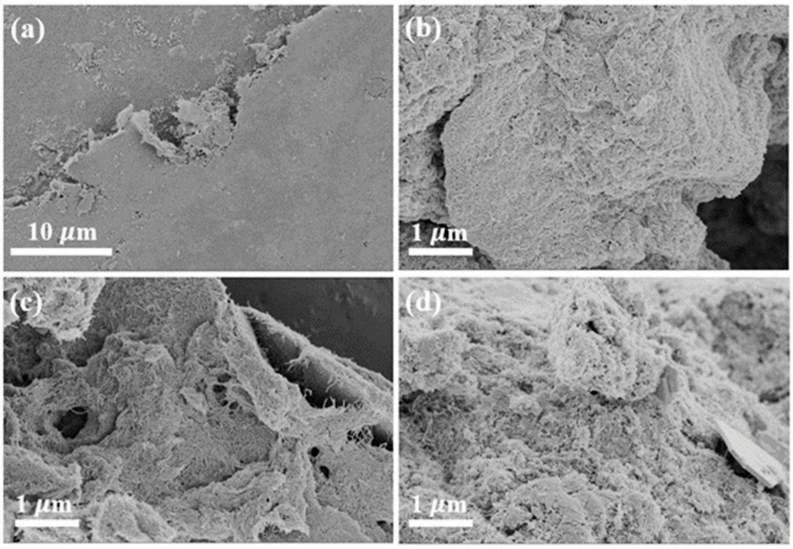

Figure S1. SEM images of **(a)** CNT, **(b)** CZL_CNT, **(c)** PN_CZ_CNT and **(d)** PN_CZL_CNT.

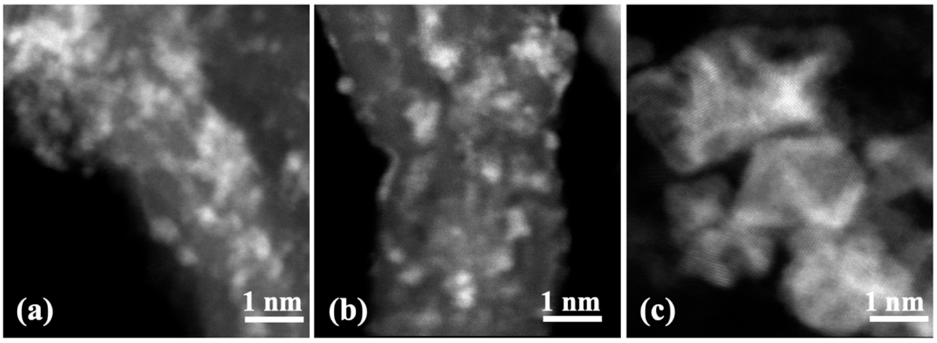

Figure S2. Dark field TEM images of **(a)** CZL_CNT, **(b)** PN_CZ_CNT and **(c)** PN_CZL_CNT.

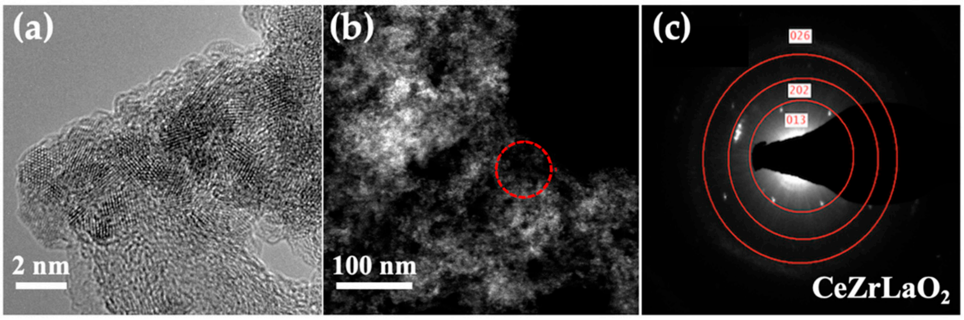

Figure S3. (a) HRTEM image of the catalyst CZL_CNT showing the lattice structure of the catalyst. **(b)** DF-TEM highlighting crystalline nanoparticles dispersed on the CNTs, circled region indicating area selected for SAED, and **(c)** SAED pattern of the circled region showing diffraction rings indexed to the $CeZrLaO_2$ support.

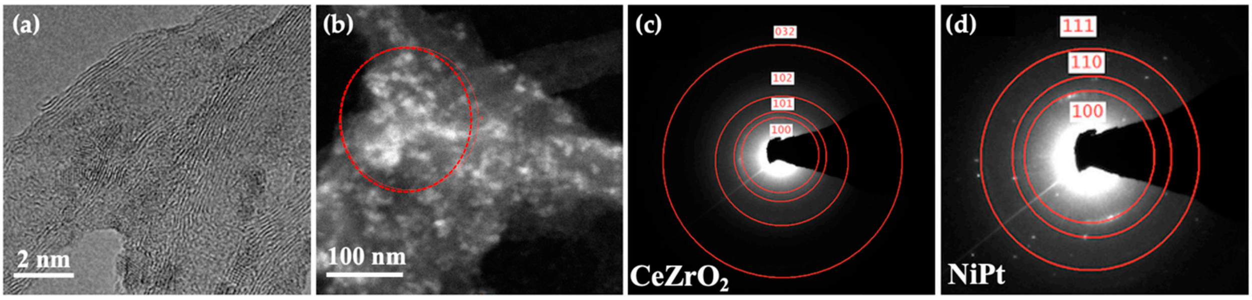

Figure S4. (a) HRTEM image of the catalyst PN_CZ_CNT showing the lattice structure of the catalyst **(b)** DF-TEM highlighting crystalline nanoparticles dispersed on the CNTs, with the circled region indicating the area selected for SAED, **(c)** SAED pattern of the circled region showing diffraction rings indexed to the $CeZrO_2$ support and **(d)** SAED pattern of the circled region showing diffraction rings attributed to Ni-Pt containing phases.

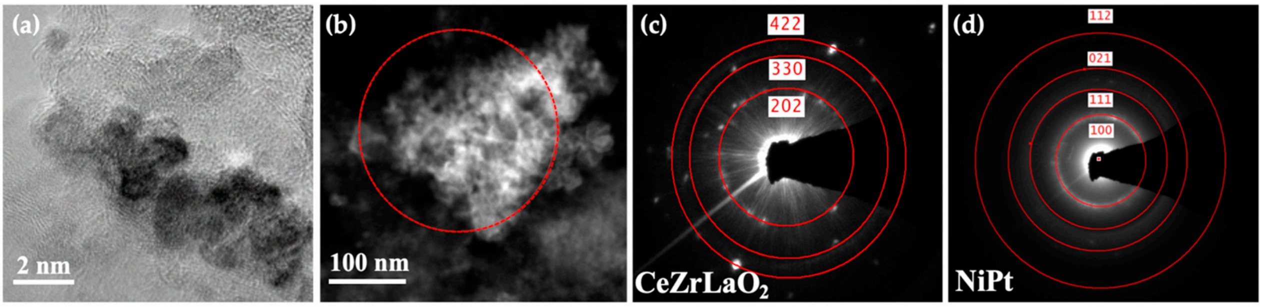

Figure S5. (a) HRTEM image of the catalyst PN_CZL_CNT showing the lattice structure of the catalyst **(b)** DF-TEM highlighting crystalline nanoparticles dispersed on the CNTs, with the circled region indicating the area selected for SAED, **(c)** SAED pattern of the circled region showing diffraction rings indexed to the $CeZrLaO_2$ support and **(d)** SAED pattern of the circled region showing diffraction rings attributed to Ni-Pt containing phases.

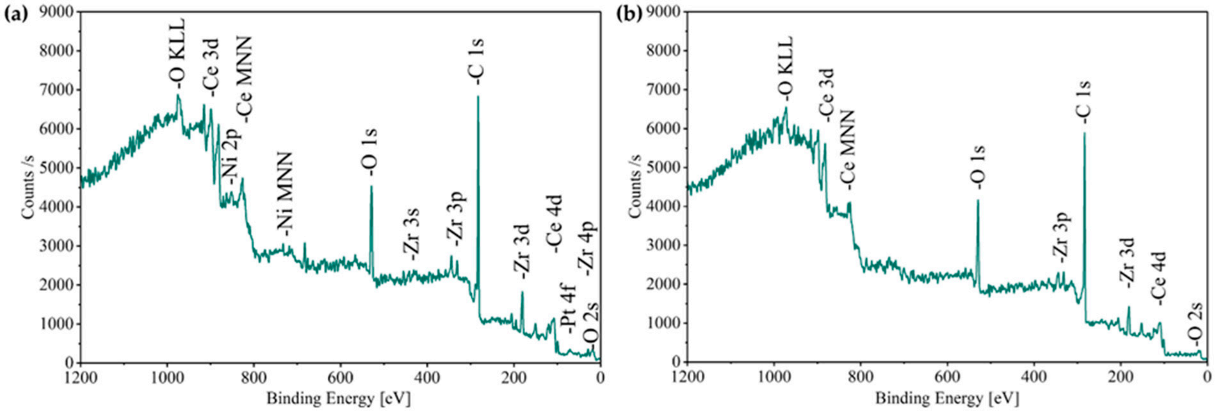

Figure S6. XPS survey spectra of **(a)** PN_CZ_CNT and **(b)** PN_CZL_CNT catalysts.

Synthesis of Ni_CNT, Ni_CZ and PN_CZL catalysts

The Ni_CNT and Ni_CZ_CNT catalysts were synthesized according to the procedure described in [1] and Ni_CZ catalyst was synthesized according to the procedure described in [2]. The PNL_CZ catalyst was synthesized by incipient wetness impregnation of the $Ce_{0.55}Zr_{0.4}La_{0.05}O_2$ (supplied by Rhodia Catalyst & Electronics, France) using Pt and Ni precursors dissolved in acetone-water mixtures (1:1 and 1:3, v/v, respectively). First, the 0.0535 g of bis(acetylacetonato) platinum(II) ($Pt(acac)_2$) was dissolved in 0.5 ml of solvent and 1.16 g of nickel(II) bis(acetylacetonate) ($Ni(acac)_2$) was dissolved in 2 ml of solvent. The precursor solutions were subsequently mixed in a single beaker and added dropwise to 5 g of $CeZrLaO_2$ under continuous stirring until complete impregnation was achieved. The impregnated material was dried at 120°C for 24 h and calcined at 800°C for 5 h to obtain the final PNL_CZ catalyst.

Table S1. Nominal loading of metals in obtained CNT-supported catalysts.

Catalyst	Nominal Composition (wt.%)						
	CNTs	Ce	Zr	La	Ni	Pt	O
**Ni_CZ**[1]	0	50.9	20.4	0	10	0	18.7
**PNL_CZ**[2]	0	47.8	22.6	4.3	5	0.5	19.8
**Ni_CNT**	90.1	0	0	0	9.9	0	0
**CZ_CNT**	80.2	12.2	3.6	0	0	0	4
**Ni_CZ_CNT**	66.0	15.5	4.8	0	8.5	0	5.2
**PN_CZ_CNT**	75.7	7.6	2.3	0	4.8	0.5	9.1
**CZL_CNT**	57.4	20	11.3	2.2	0	0	9.01
**PN_CZL_CNT**	40.3	23.8	11.3	2.2	8.8	4.6	9.01

Table S2. Average metal loading in CNT-supported catalysts determined by energy dispersive X-ray spectroscopy (EDS).

Catalyst	Composition (wt.%)					
	CNTs	Ce	Zr	La	Ni	Pt
**PN_CZ_CNT**	51.8	21.7	13.5	0	13	0
**CZL_CNT**	47.3	32.4	17.1	3.1	0	0
**PN_CZL_CNT**	41	15.6	23.4	2	9.2	8.6

Table S3. Surface composition (atomic %) of C and O in catalysts determined by XPS.

	C						O		
**Binding Energy (eV)**	**284.4**	**285.0**	**286.2**	**287.4**	**288.8**	**290.9**	**529.9**	**531.6**	**532.9**
**Ox. State/ compound**	C=C sp[2]	C-C sp[3]	C-O-C; C-OH	C=O; O-C-O	O-C=O	Shake-up	O-M[3]	O-M; O=C	O-C; -OH
**PN_CZ_CNT**	46.0	14.9	4.4	1.8	1.4	7.0	8.6	4.0	5.5
**PN_CZL_CNT**	51.0	13.9	3.5	1.9	2.0	5.9	8.4	4.4	5.3

[1] Ni_CZ was prepared using $Ce_{0.62}Zr_{0.38}O_2$ support supplied by Rhodia Catalysts.

[2] PNL_CZ was prepared using $Ce_{0.55}Zr_{0.4}La_{0.05}O_2$ support supplied by Rhodia Catalysts.

[3] M=Metal

Table S4. Surface composition (atomic %) of metals in catalysts determined by XPS.

	Ce		Zr	Ni	Pt	La
**Binding Energy (eV)**	**882.1 884.7**	**882.8 886.6 898.6**	**182.3**	**855.7**	**71.4**	**----**
**Ox. State/ compound**	Ce^{3+}	Ce^{4+}	Zr^{4+}	Ni^{2+}	Pt(0)	----
**PN_CZ_CNT**	0.3	1.6	2.0	----	0.04	----
**PN_CZL_CNT**	0.4	2.0	1.4	----	----	----

$$\text{Carbon Balance (\%)}: \frac{[CH_4]_{outlet} + [CO_2]_{outlet} + [CO]_{outlet}}{[CH_4]_{inlet} + [CO_2]_{inlet}} \times 100 \quad \text{(Eq. S1)}$$

$$\text{Hydrogen Balance (\%)}: \frac{2\times[CH_4]_{outlet} + [H_2]_{outlet}}{2\times[CH_4]_{inlet}} \times 100 \quad \text{(Eq. S2)}$$

Table S5. Carbon balance calculated from Eq. S1 during catalytic tests of DRM (4 vol.% CH_4, 10 vol.% CO_2, balanced with Ar; TOS = 2 h, GHSV = 10000 1/h).

Carbon balance (%)							
**T (°C)**	**Ni_CZ**	**PNL_CZ**	**Ni_CNT**	**CZ_CNT**	**Ni_CZ_CNT**	**PN_CZ_CNT**	**PN_CZL_CNT**
**450**	91.9	-	100	-	-	-	-
**500**	89.9	97.5	100	100	100	-	-
**550**	85.2	-	100	99.8	100	-	-
**600**	81.1	96.3	100	99.7	100	94.7	-
**650**	78.7	-	100	99.9	100	-	-
**700**	79.1	94.0	100	100	100	92.6	74.8
**750**	77.0	-	100	100	100	-	-
**800**	74.9	91.8	100	99.9	100	98.2	80.2
**850**	-	-	100	100	100	-	-
**900**	-	-	100	100	100	-	-

Table S6. Hydrogen balance calculated from Eq. S2 during catalytic tests of DRM (4 vol% CH_4, 10 vol% CO_2, balanced with Ar; TOS = 2 h, GHSV = 10000 1/h).

Hydrogen balance (%)							
**T (°C)**	**Ni_CZ**	**PNL_CZ**	**Ni_CNT**	**CZ_CNT**	**Ni_CZ_CNT**	**PN_CZ_CNT**	**PN_CZL_CNT**
**450**	90.6	-	99.8	-	-	-	-
**500**	94.6	99.6	98.9	100	100	-	-
**550**	95.5	-	98.2	100	99.8	-	-
**600**	94.0	88.1	96.4	100	99.8	91.0	-
**650**	92.8	-	96.5	100	98.5	-	-
**700**	91.0	85.7	95.2	100	97.3	86.3	65.2
**750**	93.0	-	94.0	100	97.7	-	-
**800**	92.1	81.0	93.0	100	96.2	82.3	64.3
**850**	-	-	92.7	100	96.3	-	-
**900**	-	-	92.7	100	95.7	-	-

References:

1. Łamacz, A.; Jagódka, P.; Stawowy, M.; Matus, K. Dry Reforming of Methane over CNT-Supported CeZrO2, Ni and Ni-CeZrO2 Catalysts. *Catalysts* **2020**, *10*, 741, doi:10.3390/catal10070741.
2. Łamacz, A.; Pawlyta, M.; Dobrzański, L.A.; Krztoń, A. Characterization of the Structure Features of CeZrO2 and Ni/CeZrO2 Catalysts for Tar Gasification with Steam. *Archives of Materials Science and Engineering* **2011**, *48, 89-96*.
